# Endoscopic ultrasonography-guided rendezvous and fine-caliber multi-hole metal stent for postoperative pancreatic anastomotic stricture

**DOI:** 10.1055/a-2825-8615

**Published:** 2026-03-24

**Authors:** Tatsuya Kurokawa, Hirotsugu Maruyama, Yuji Kawata, Yoshinori Shimamoto, Yuki Ishikawa-Kakiya, Kojiro Tanoue, Yasuhiro Fujiwara

**Affiliations:** 1 Department of Gastroenterology, Graduate School of Medicine, Osaka Metropolitan University, Osaka, Japan

**Fig. 1:**
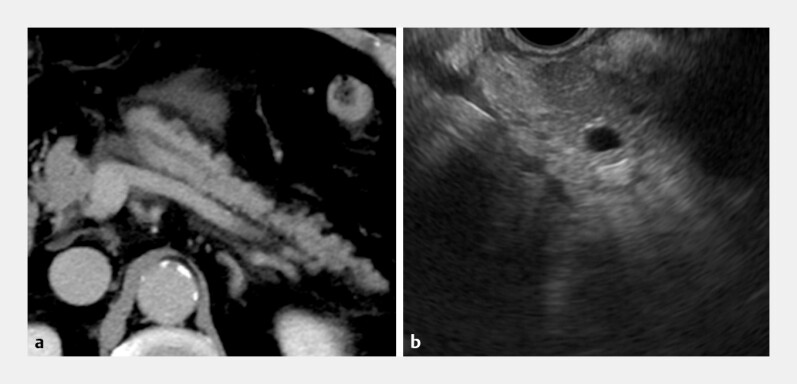
Preprocedural imaging. **a** Contrast-enhanced computed tomography showing pancreatic duct dilatation distal to the anastomosis with peripancreatic fluid collection consistent with acute pancreatitis. **b** Endoscopic ultrasonography demonstrating a dilated pancreatic duct distal to the anastomosis.

**Fig. 2:**
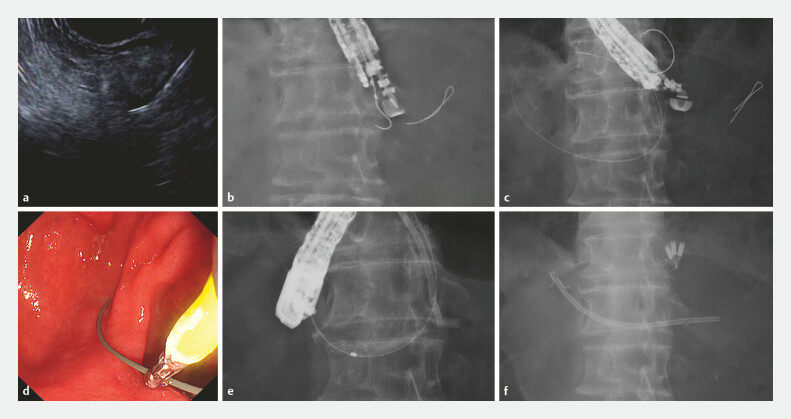
EUS-guided rendezvous placement of a plastic pancreatic stent across the anastomotic stricture. **a** Pancreatography after puncture showing the dilated pancreatic duct distal to the anastomosis. **b** A guidewire advanced into the stomach via the anastomosis. **c** Placement of a plastic pancreatic stent across the anastomotic stricture using the rendezvous approach. **d** The 0.025-inch guidewire was grasped with biopsy forceps and carefully drawn into the working channel. **e** With the rendezvous technique, a catheter was advanced across the anastomosis over the guidewire. **f** A plastic pancreatic stent was placed across the anastomosis. EUS, endoscopic ultrasonography.

**Fig. 3:**
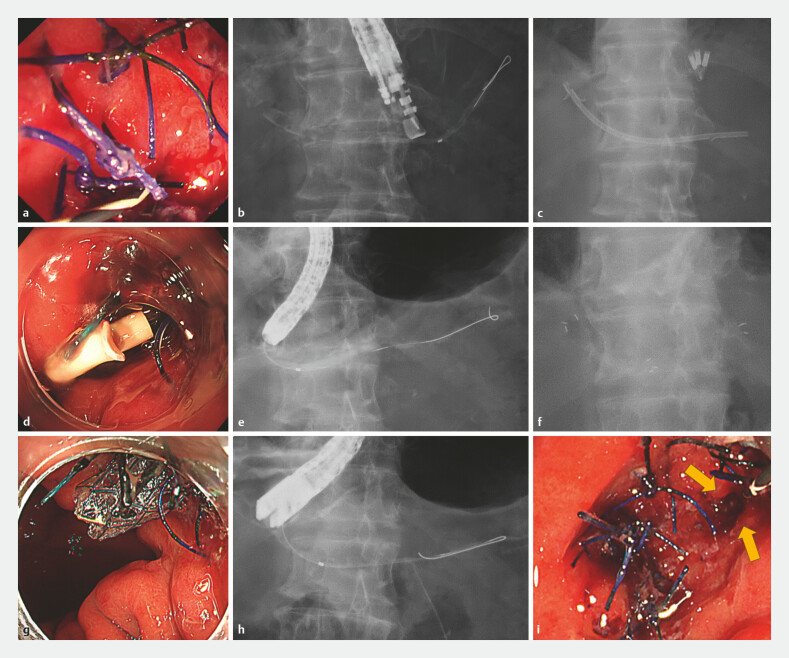
Serial endoscopic and fluoroscopic findings during treatment. **a** An endoscopic view of the stenotic postoperative anastomosis. **b** Pancreatography demonstrating upstream pancreatic duct dilatation. **c** A fluoroscopic image showing plastic stent placement across the anastomosis. **d** An endoscopic view after plastic stent placement across the anastomosis. **e** Pancreatography after plastic stent removal showing improved pancreatic duct dilatation. **f** A fluoroscopic image showing the deployment of the MHSEMS across the anastomosis. **g** An endoscopic view after MHSEMS deployment across the anastomosis. **h** Pancreatography after MHSEMS removal showing the resolution of pancreatic duct dilatation. **i** An endoscopic view of the patent anastomosis after MHSEMS removal.

Endoscopic treatment for postoperative pancreatic anastomotic strictures is challenging 1 2 . Difficulty in identifying the anastomotic site and the need for repeated plastic stent exchanges further complicate treatment 2 . In such cases, treatment that ensures reliable access to the anastomotic site and directly improves the stricture itself is required 3 4 . Here, we report a case in which an endoscopic ultrasonography (EUS)-guided rendezvous technique successfully secured pancreatic duct access, enabling the effective treatment of a postoperative anastomotic stricture using a novel fine-caliber, multi-hole self-expandable metal stent (MHSEMS). A 73-year-old man who had undergone middle pancreatectomy with gastro-pancreatic anastomosis 15 years earlier presented with abdominal pain. Computed tomography suggested pancreatitis due to an anastomotic stricture ( Fig. 1 ). Pancreatic duct stenting was unsuccessful, and he was referred to our hospital. An EUS-guided rendezvous procedure was performed. Using a 22-gauge FNA needle, the dilated distal pancreatic duct was punctured, and a 0.018-inch guidewire was advanced distally. A double-lumen catheter was used to redirect a 0.025-inch guidewire across the anastomosis and into the stomach. A duodenoscope (TJF-290; Olympus, Tokyo, Japan) was then used to complete the rendezvous and placed a 7 Fr plastic pancreatic stent ( Fig. 2 ). One month later, the plastic stent was exchanged for an MHSEMS (6 mm × 8 cm; Hanarostent Biliary Multi-Hole Benefit; M.I. Tech, Pyeongtaek, South Korea) using a therapeutic gastroscope (GIF-290T; Olympus, Tokyo, Japan). Two months later, the MHSEMS was removed easily using grasping forceps with a duodenoscope ( Video 1 and Fig. 3 ). The anastomosis remained patent, and the patient remained symptom-free 5 months after stent removal.

EUS-guided rendezvous and MHSEMS dilation for the postoperative pancreatic anastomotic stricture. EUS, endoscopic ultrasonography.Video 1

This 6 mm MHSEMS achieved effective dilation of a postoperative pancreatic anastomotic stricture. Its design could reduce the risks of pancreatitis and migration. The combination of the EUS-guided rendezvous technique allows more reliable treatment of pancreatic duct anastomotic strictures.

Endoscopy_UCTN_Code_TTT_1AS_2AI
